# Indispensable role of β-arrestin2 in the protection of remifentanil preconditioning against hepatic ischemic reperfusion injury

**DOI:** 10.1038/s41598-018-38456-9

**Published:** 2019-02-14

**Authors:** Yuting Yang, Caiyang Chen, Cui Cui, Yingfu Jiao, Peiying Li, Ling Zhu, Weifeng Yu, Qiang Xia, Daxiang Wen, Liqun Yang

**Affiliations:** 1 Department of Anesthesiology, Renji Hospital, Shanghai Jiao Tong University School of Medicine, Shanghai, China; 2 Department of Anesthesia and Intensive Care, Eatern Hepatobiliary Surgery Hospital, Second Military Medical University, Shanghai, China; 3 Department of Hepatic Surgery, Renji Hospital, Shanghai Jiao Tong University School of Medicine, Shanghai, China

## Abstract

Our previous study demonstrated that remifentanil, an opioid agonist, conferred profound liver protection during hepatic ischemia reperfusion injury (HIRI), in which Toll-like receptors (TLRs) played a crucial role in mediating the inflammatory responses. β-arrestin2, a well-known mu opioid receptor desensitizer, is also a negatively regulator of Toll-like receptor 4 (TLR4)-mediated inflammatory reactions in a mitogen-activated protein kinase (MAPK)-dependent manner. Using the rodent models of hepatic ischemia reperfusion injury both in wild type and TLR4 knockout (TLR4 KO) mice, we found that remifentanil preconditioning could inhibit the expression of TLR4 and reduce the inflammatory response induced by HIRI in wild type but not in TLR4 KO mice. For the *in-vitro* study, LPS was used to treat RAW264.7 macrophage cells to mimic the inflammatory response induced by HIRI. Remifentanil increased β-arrestin2 expression both *in vivo* and *in vitro*, while after silencing β-arrestin2 RNA, the effect of remifentanil in reducing cell death and apoptosis, as well as decreasing phosphorylation of ERK and JNK were abolished in RAW264.7 cells. These data suggested that remifentanil could ameliorate mice HIRI through upregulating β-arrestin2 expression, which may function as a key molecule in bridging opioid receptor and TLR4 pathway.

## Introduction

Hepatic ischemia-reperfusion injury (HIRI), is a severe complication of many clinical settings, which can lead to liver cell death, function loss, so that greatly impairs patients’ future prognosis and is often inevitable during liver surgeries and systemic circulatory disturbances^1^. Even though the underlying mechanisms of IR injury are still poorly clarified, previous publications hold that toll-like receptors (TLRs) are widely described as key regulators of the inflammation and hepatic injury involved in HIRI, and the activation of TLRs on Kupffer cells (KCs) may play a fundamental role in the process^2^. Toll-like receptor 4 (TLR4) stimulation during IR initiates a number of intracellular signaling events which ultimately activate mitogen-activated protein kinase (MAPK) cascades and nuclear factor kappa B and thereby induce the inflammatory reactions after reperfusion^3^.

Remifentanil, an ultra-short-acting opioid receptor agonist, has universal protective effect against ischemic reperfusion injury in multiple organs^4,5^. Our previous study confirmed that remifentanil can inhibit inflammatory responses against HIRI via inducible nitric oxide synthase (iNOS) expression^6^, which in line with other researches that reported remifentanil could also alleviate hepatic apoptosis^7^. TLR4 was suggested to mediate inflammatory responses which contributes to organ ischemia and reperfusion injury^8^. However, how does the opioid agonist inhibit TLR4 and therefore provide inhibitory effect on inflammatory response after HIRI remains remarkably unknown. The cytosolic scaffold and signaling protein β-arrestin2 was shown to act as a negative regulator of LPS-induced TLR4 activation^9^. Furthermore, accumulating evidences indicated that β-arrestin plays an important role not only in the phosphorylation and internalization of opioid receptor, but also in the modulation of immune responses by binding to TRAF6^10,11^. Since mu opioid receptor binds to β-arrestin2 with higher affinity than other members of β-arrestins family^12^, we hypothesized that β-arrestin2 was an important link between remifentanil preconditioning and TLR4 related inflammation.

The objective of our study was to investigate the involvement of β-arrestin2 in the anti-inflammatory effect of remifentanil preconditioning during HIRI in mice. Here, we planned to confirm that remifentanil preconditioning inhibits the expression of TLR4, attenuates inflammatory response in HIRI and confers protection against HIRI. Using β-arrestin2 targeted siRNA, we expect to address that β-arrestin2 is the key molecule bridging opioid receptor and TLR4 pathway and played indispensable role in the remifentanil preconditioning afforded protection against HIRI. Considering that remifentanil preconditioning is an easily accessible therapeutic paradigm, elucidating the mechanism underlying its protective effect may further pave the way for its translation into clinical practice.

## Results

### Remifentanil preconditioning down-regulated TLR4 expression, attenuated inflammatory response and liver injury after ischemia and reperfusion

**Figure 1:**
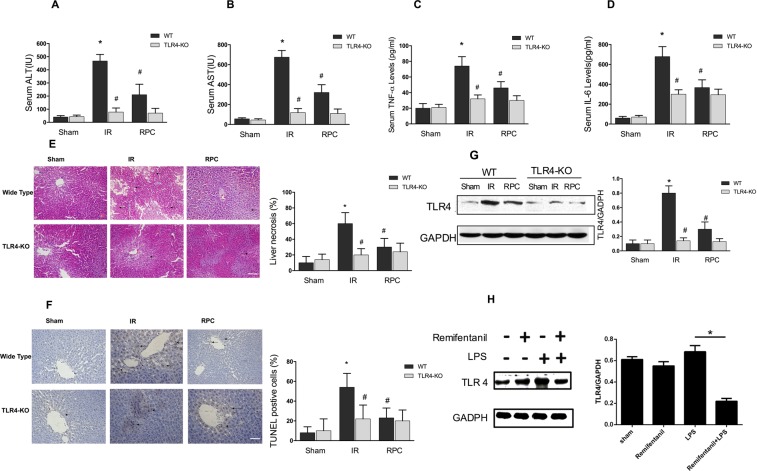
(**A**–**D**) In WT mice, the levels of serum aminotransferases (ALT and AST), TNF-α and IL-6 increased significantly in IR group compared with sham group, and decreased in RPC group compared with IR group (*P < 0.05, vs Sham, ^#^P < 0.05, vs IR); In TLR4 KO mice, there were no significant differences between RPC group and IR group. (**E**,**F**) Hepatic tissue histological injury extent was detected with H&E staining for light microscopy examination. The area in the photograph signed with black arrows depicted typical pattern of focal necrosis resulted by ischemia. Hepatocyte apoptosis was determined by TUNEL method for immunohistochemical. The area in the photograph signed with black arrows depicted apoptotic positive cells. In WT mice, Areas of necrosis (Magnification: 200×, Scale bar = 200 μm) and percentage of apoptotic cells (Magnification: 400×, Scale bar = 100 μm) were both significantly lower in the RPC group than in the IR group. In TLR4 KO mice, there were no significant difference in liver necrosis and apoptosis between the RPC group and IR group. (**G**) Western blot analysis of TLR4 protein expression in liver tissues in the wild type and TLR4 KO mice. GAPDH was used as the loading control. (**H**) RAW264.7 cells were pretreated with 10 ng/ml remifentanil for 1 h and then stimulated by 1 μg/ml LPS for another 6 h, the protein expression of TLR4 was measured by western blot. *P < 0.05 compared to LPS alone. (n = 8 in each group and 3 repeats of each experiment).

Emerging evidences suggest that remifentanil preconditioning may confer protection against ischemia reperfusion injury in multiple organs^4,5^. The inflammatory cytokine serum tumor necrosis factor-α (TNF-α) and interleukin-6 (IL-6) concentrations decreased significantly 2 h after reperfusion in the RPC (Remifentanil Preconditioning) group when compared with the IR (Ischemia Reperfusion) group (Fig. 1C,D). In addition, we found that remifentanil preconditioning provided robust protection against hepatic IR injury as determined by serum aminotransferases (ALT and AST, Fig. 1A,B) measurement at 2 hours after surgery, while the liver tissues of RPC group showed less pathological injury as compared with the IR group (Fig. 1E). The amount of apoptosis and the number of terminal deoxynucleotidyl transferase mediated-deoxyuridine triphosphate nick end labeling (TUNEL) positive cells in the RPC group were also less than that in the IR group (Fig. 1F). Interestingly, the above mentioned hepatic protective and anti-inflammatory effects of remifentanil preconditioning were observed only in wild type (WT) but not TLR4 KO mice (Fig. 1A–F). TLR4 is increasingly reported to mediate inflammatory responses which contributes to organ IR injury^13^. Therefore, we next examined the TLR4 expression after hepatic IR injury with or without remifentanil treatment. The TLR4 expression was robustly increased after IR injury without remifentanil treatment. However, the TLR4 expression of wild type mice was significantly inhibited in the RPC group (Fig. 1G). To further detect the anti-inflammatory effect of remifentanil preconditioning *in vitro*, 1 μg/ml of LPS was used to treat raw264.7 cells to mimic HIRI which mediated by inflammatory response of KCs and we found that the level of TLR4 protein expression in the cells treated with LPS and remifentanil was much lower than in the cells treated with LPS only (Fig. 1H), indicating remifentanil could possibly down-regulate TLR4 expression. Collectively, these data suggested that remifentanil preconditioning down-regulated TLR4 expression and exerted anti-inflammatory effect during HIRI.

### Remifentanil pretreatment increases β-arrestin2 expression both in liver tissue of mice subjected to IR and RAW 264.7 cells

**Figure 2:**
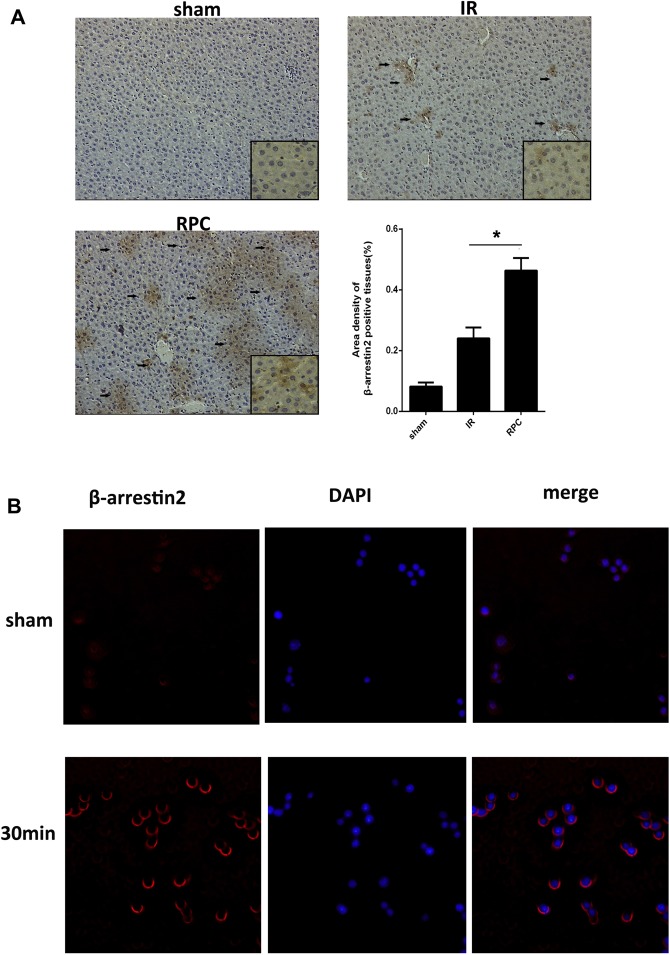
The expression of β-arrestin2 in mice liver tissue and RAW264.7 macrophage cells culture with remifentanil pretreatment. (**A**) Hepatic β-arrestin2 positive cells were defined as stained with brown in cytoplasm (black arrows), there was an increase expression of β-arrestin2 in RPC groups compared with those in IR groups. (magnification: 200×, 400×; *P < 0.05 compared with IR, n = 8 in each group). (**B**) RAW264.7 cells were pretreated with 10 ng/ml remifentanil for 30 min and β-arrestin2 expression was measured by immunoflurencence. Remifentanil pretreatment could upregulate the expression of β-arrestin2 (magnification, 400×). All the results were from at least three independent experiments.

Mounting evidence indicates that the negative regulation of β-arrestin2 can be involved in LPS-induced TLR4 activation^9^ and inflammation^14^. However, it is unknown whether remifentanil have an effect on the expression of β-arrestin2 to regulate the inflammatory response induced by HIRI. As shown in Fig. 2A,the expression of β-arrestin2 was significantly higher in RPC group compared with IR group and sham group (P < 0.05) in liver. The β-arrestin2 protein was observed mainly located in the cytoplasm(Fig. 2A). Furthermore, *in vitro* experiment, immunofluorescence staining was used to detect the expression of β-arrestin2 in RAW264.7 cells. The β-arrestin2 expression in the plasma membranes was dramatically improved after preincubation with 10 ng/ml remifentanil for 30 min. However, in the sham group, β-arrestin2 mainly located in the cytoplasm (Fig. 2B). These data indicated that remifentanil pretreatment could upregulate the expression of β-arrestin2, and even mediate the redistribution of β-arrestin2 expression from the cytoplasm to the cell membrane.

### Effect of remifentanil pretreatment on RAW264.7 cells viability and apoptosis induced by LPS with β-arrestin2 inhibition

**Figure 3:**
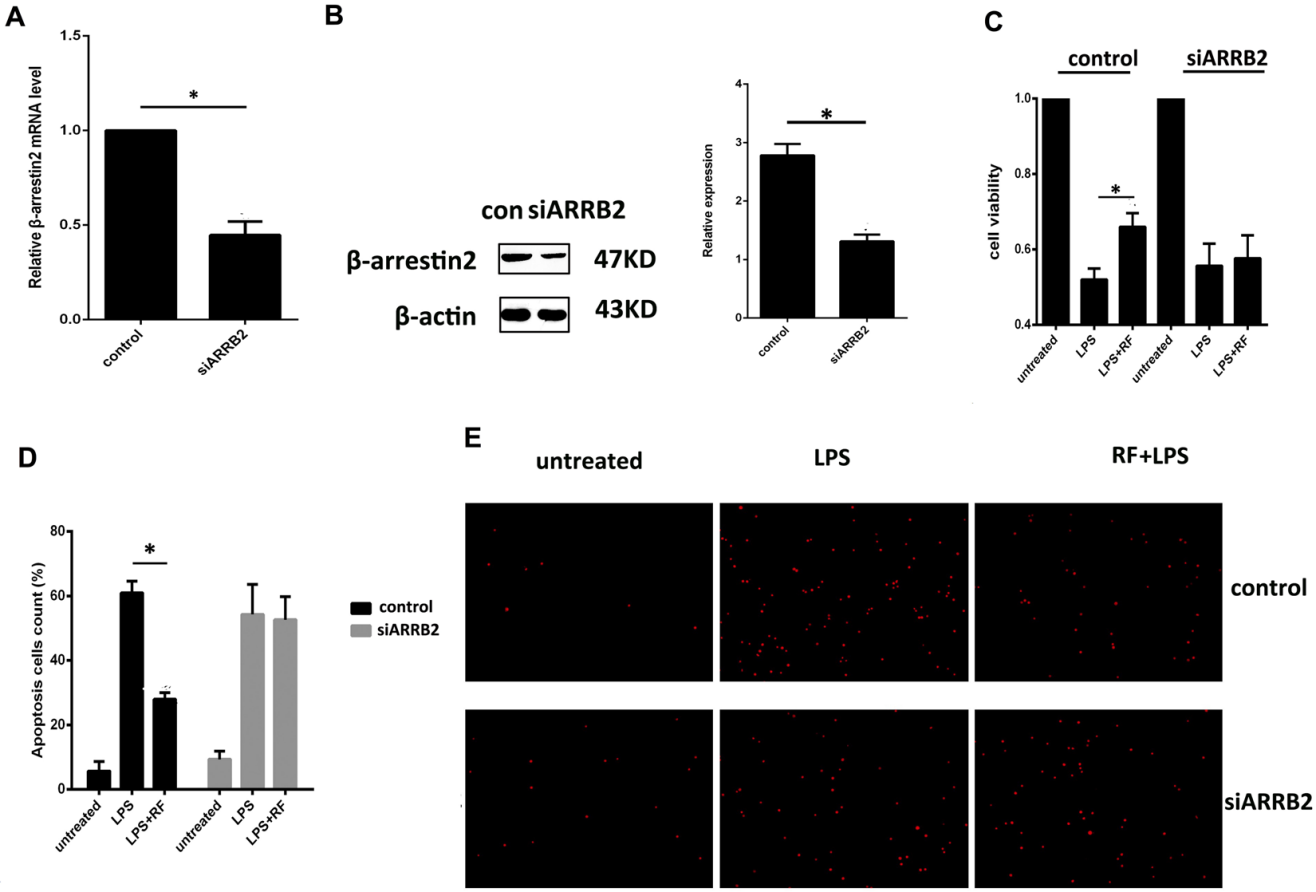
Effects of β-arrestin2 on remifentanil pretreatment of RAW264.7 cells viability and apoptosis induced by LPS. (**A**) The expression of β-arrestin2 mRNA was measured by RT-PCR, which revealed that β-arrestin2 mRNA decreased about 60% after transfected with β-arrestin2 siRNA. *P < 0.05 compared to control. (**B**) The expression of β-arrestin2 protein was measured by western blot, which revealed that β-arrestin2 protein decreased about 55% after transfected with β-arrestin2 siRNA. *P < 0.05 compared to control. (**C**) Cell viability increased by remifentanil (10 ng/ml) pretreatment, measured by WST-8 dye, whereas the effect was abolished when silencing β-arrestin2 expression with siRNA. (*P < 0.05 compared to LPS alone). (**D**,**E**) Cell apoptosis was assessed by Hoechst 33342 dye assay, the nuclei at the early stage of apoptosis displayed an increased brightness of chromatin stain. Cell apoptosis stimulated by LPS (1 μg/ml) was attenuated after remifentanil pretreatment, whereas the effect was blocked when silencing β-arrestin2 expression with siRNA. (magnification: 200×; *P < 0.05 compared to LPS alone). All the results were from at least three independent experiments.

To further investigate the role of β-arrestin2 in HIRI afforded by remifentanil pretreatment, siRNA targeting β-arrestin2 mRNA was constructed to interfere the expression of β-arrestin2 in RAW264.7 cells. As shown in Fig. 3A, RT-PCR was used to observe that there was about 65% decrease of β-arrestin2 mRNA in the interference group as compared with control group. In addition, the western blot results revealed that the expression of β-arrestin2 protein in the interference group declined about 50% over the control group (Fig. 3B).

Cell proliferation ability was assessed by cell counting kit-8 (CCK8) colorimeter, which revealed that in the control group, cell viability decreased about 50% after treated with 1 μg/ml LPS for 24 h, and retained up to 65% in the group pretreated with remifentanil (Fig. 3C). However, there was no significant difference between groups pretreated with or without remifentanil in group of RAW264.7 transfected with β-arrestin2 targeting siRNA. Cell apoptosis induced by LPS stimulation was significantly attenuated by remifentanil pretreatment. But this alleviating effect was also abolished in β-arrestin2 silenced cells (Fig. 3D,E). Taken together, above-mentioned data presented here supported a critical role of β-arrestin2 in the protection of remifentanil against cell apoptosis and cell death of macrophages following LPS challenge.

### Effects of remifentanil on the phosphorylation of ERK and JNK and the formation of β-arrestin2 and TRAF6 in RAW264.7 cells

**Figure 4:**
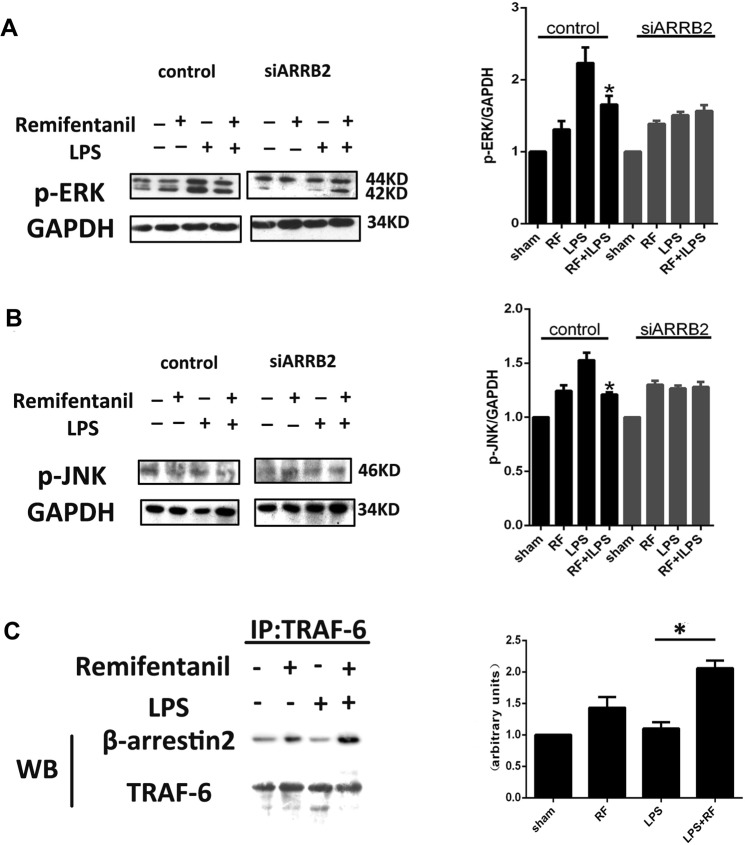
Effects of remifentanil on the JNK/ERK1/2 activity and β-arrestin2-TRAF6 complex formation induced by LPS stimulation in RAW264.7 cells. (**A**,**B**) Cells were pretreated with remifentanil (10 ng/ml) for 1 h before 6 h of LPS (1 μg/ml) stimulation. Deficiency of β-arrestin2 abolished the inhibitory effect of remifentanil on phosphorylation of ERK1/2 and JNK induced by LPS stimulation. Changes in phospho-ERK1/2 and JNK are expressed as ratios of phosphorylated kinases/GAPDH are shown as a bar diagram (*P < 0.05 compared to LPS alone). (**C**) Cells were pretreated with remifentanil (10 ng/ml) for 30 min, then were stimulated by LPS (1 μg/ml) for another 30 min. TRAF6 was immunoprecipitated and associated β-arrestin2 was analyzed by Western blot. All the results were from at least three independent experiments.

Previous study has demonstrated that β-arrestin 2 negatively regulated TLR4-mediated inflammatory reactions in a MAPK-dependent mechanism. The phosphorylation and activation of the key molecules of MAPKs, such as ERK and JNK has been shown to function as the downstream regulators of TLR4 signaling in response to several types of stresses including HIRI^15–17^. To further investigate whether remifentanil down-regulated ERK and JNK activation by enhancing β-arrestin2 expression, phosphorylation of ERK and JNK in RAW264.7 cells were measured after challenged with LPS stimulation. The result of western blot showed that remifentanil reduced the phosphorylation of ERK and JNK induced by LPS, while this effect was blocked when interfering β-arrestin2 expression (Fig. 4A,B). Furthermore, previous studies have confirmed that association of β-arrestin and TRAF6 negatively regulates TLR and JNK/ERK signaling and subsequently down-regulate inflammatory responses^10^, therefore, the formation of β-arrestin2-TRAF6 complex could be crucial for remifentanil mediated anti-inflammatory effect. In present study, we found that remifentanil preconditioning could increase the amount of β-arrestin2-TRAF6 complex (Fig. 4C). These results indicated that phosphorylation of ERK and JNK and formation of β-arrestin2/TRAF6 complex might underlie the crosstalk between remifentanil preconditioning and inhibiting of TLR4 inflammatory pathway.

## Discussion

The present study characterizes the important role of β-arrestin-2 in the remifentanil preconditioning afforded protection against HIRI. Remifentanil, as an ultra-short-acting opioid analgesic agent, is favorably used during series kinds of surgeries^18^ because of its titratable pharmacokinetic profile that is beneficial to postoperative recovery. Emerging evidences suggest that it has protective effects against ischemic injury in multiple organs, such as heart and kidney in rats^19,20^. Our previous study confirmed that remifentanil preconditioning alleviated HIRI by modulating iNOS expression. In this study, we show that remifentanil preconditioning inhibited TLR4 expression and reduced inflammatory responses in HIRI. The protective effect of remifentanil was examined by measuring serum aminotransferases concentrations, a widely accepted marker of hepatocyte injury. In addition, we also detected a number of histologic and biochemical variables to evaluate hepatic injury more thoroughly and found that remifentanil pretreatment group is associated with less cell death and tissue loss.

KC is the major source of oxidative stress substrates in response to ischemic injury^21,22^, releasing inflammatory cytokines, such as TNF-α and IL-β, which contributes to ischemic hepatocytes injury. It’s well known that LPS can induce TLR4 activation followed by inflammatory cytokine release^23,24^, which also results in KCs activation and hepatic inflammatory injury^25^. We found that remifentanil pretreatment can reduce the level of inflammatory cytokines TNF-α and IL-6 and promote hepatocyte proliferation after hypoxia/reoxygenation injury in the co-culture system of hepatocyte-KC based on the supplementary experiments, which provided a further indication for remifentanil hepatic protection. We used LPS to stimulate RAW264.7 cells and induce macrophages damage *in vitro*, our results suggested that remifentanil also inhibited TLR4 expression induced by LPS stimulation which was in accord with another opioid agonist study in nervous system^26^. Likewise, there are evidences from other research groups showing that morphine can suppress TLR4 protein and mRNA expression in macrophages by activating mu opioid receptor^27^. Collectively, all the above evidences indicate that there is internal relationship between mu opioid receptor and TLR4, and the interaction between mu opioid receptor and TLR4 may have a pivotal role in the remifentanil mediated hepatic protection.

β-arrestin family is composed of visual arrestin, β-arrestin1 and β-arrestin2. The classical function of β-arrestin is regulating endocytosis and desensitization of GPCR, and some non-GPCR desensitization^28,29^. In addition, β-arrestin participates in the immune regulation process. There are evidences showing that both β-arrestin1 and β-arrestin2 are increased following cerebral ischemia. β-arrestin1 has been found to have neuroprotective effects against ischemia^30^ and β-arrestin2 was reported to be involved in the transduction of extracellular to intracellular signals as a signaling protein molecules. It has been confirmed that β-arrestin2 could reduce LPS-induced TLR4 signaling *in vitro* and vivo experiments^31,32^. Wang *et al*. demonstrated that β-arrestin2 negatively regulated LPS-induced TLR4 signaling pathway, reducing the expression of inflammatory cytokines and endotoxin shock in mice. β-arrestin2 interacts with JNK3 isoforms and apoptosis signaling kinase 1 (ASK1), and protects cells from apoptosis induced by hypoxia^33^. In this study, we found that remifentanil pretreatment increased β-arrestin2 expression in liver tissue following IR injury. Similarly, the expression of β-arrestin2 in RAW264.7 cells was reregulated after remifentanil preconditioning, and we found a tendency of the protein to gather to the membrane from the cytosol, indicating that β-arrestin2 may play an important role in remifentanil pretreatment.

To further determine the role of β-arrestin2, siRNA was used to interfere β-arrestin2 expression in RAW264.7 cells. During ischemia reperfusion, LPS acts as one of the main factors that cause cell apoptosis and necrosis, and we confirmed that remifentanil could relieve cell apoptosis induced by LPS stimulation, while this protective effect was abolished when β-arrestin2 was silenced by siRNA. Therefore, promoting β-arrestin2 activation may be a possible mechanism underlying remifentanil’s liver protection.

ERK and JNK are key molecules of MAPKs, which located in downstream of TLR4 and MyD88 pathways, and is a key downstream regulator of TLR signaling^34,35^. It was also found that β-arrestin2 regulated TLR4-stimulated IL-10 response via p38-MAPK activity^3^. Consistently, our study showed that phosphorylation of ERK and JNK in RAW264.7 cells induced by LPS was blocked by remifentanil pretreatment, and interestingly, deficiency of β-arrestin2 abolished this effect, suggesting that β-arrestin2 contributed to the protection of cell function by limiting MAPK pathway activity. Moreover, we confirmed that remifentanil increased the amount of formation of β-arrestin2-TRAF6 complex, which had been reported to act as a negative regulator of TLR signaling. These data for the first time revealed the possible intrinsic link between mu opioid receptor and TLR4 signaling, and β-arrestin2 might be a critical factor.

In summary, our study suggested that β-arrestin2 mediated the TLR4 inhibition and anti-inflammatory responses conferred by remifentanil preconditioning, which attenuated liver ischemia reperfusion injury *in vivo* and protected cells from death and apoptosis *in vitro*. As a well-known mu receptor desensitizer, β-arrestin2 played indispensable role in remifentanil afforded anti-inflammatory liver protection.

## Materials and Methods

### Animals

Male C57/B6 mice (4–6 weeks, 15–20 g) (from the Animal Care and Use Committee of the second Military Medical University, Shanghai, China) were used. TLR4 knockout mice (4–6 weeks, 150 g) were bought in Model Animal Research Center of Nanjing University. Animals were housed in groups of three to four in a cage according to guidelines from the Institutional Animal Care and Use Committee (IACUC) of Renji Hospital. The protocol for animal handling and experiments was approved by Renji Hospital IACUC. Animals were allowed to take food and water freely until the night before anesthesia.

### Surgical preparation

In brief, a dose of 30 mg.kg^−1^ pentobarbitone was used for anesthesia by intraperitoneal injection, and 10 mg.kg^−1^ for supplement if necessary. A heating pad was used to maintain body temperature. The hepatic ischemia reperfusion injury model of mice was designed according to the earlier paper^36^, in which the blood supply of liver left lateral and median lobes was temporarily interrupted. After 45 minutes ischemia and 2 hours reperfusion, we collected blood samples from eyeballs for liver function and cytokine detection. The hepatic tissue samples were gathered after perfusing liver with cold saline for later experiments.

### Study groups of experiment *in vivo*

Wild type and TLR4 knockout mice were randomly divided into three groups respectively (n = 8 in each). Mice in control group received relative vessel operation but not blood interruption; liver tissue and blood samples were collected after reperfusion. Among those in IR group, liver ischemia was produced for 45 minutes as previously mentioned and followed by 2 hours reperfusion. Animal in RPC group received the same surgical procedure, and 30 μg/kg of remifentanil was intraperitoneally injected 10 minutes before the onset of liver ischemia.

### Measurement of serum aminotransferase concentration and cytokines

Serum aminotransferase including ALT and AST were measured with the aid of a special kit (Nan-Jing Jiancheng Biochemicals Ltd, China) in an automatic analyzer (Hitachi, Tokyo, Japan). Serum TNF–α, IL-6 were detected by a mouse ELISA kit (Boshide Biochemicals Ltd, Wuhan, China) according to the product reference.

### Histopathology examination and apoptotic cell detection TUNEL staining

Liver samples were prepared after 2 hours reperfusion and fixed in paraformaldehyde immediately, every sample was cut into small portions (0.5 cm × 0.5 cm) and embedded in paraffin after a series of dehydration process. These portions were performed into 4 μm thick sections and stained with hematoxylin and eosin (H&E)^37^. High-powered microscopy (1 × 200) was used to analyze the extent of liver injury. Liver tissue apoptosis was detected with TUNEL method^38^ (*In Situ* Cell Detection Kit, Roche Biochemicals, Mannheim, Germany).

### Immunohistochemistry of β-arrestin2 protein in liver tissues

The fixed liver block was embedded in paraffin and sectioned into 5 μm slices. Each liver section was deparaffinized by xylene and rehydrated with graded alcohols. After antigen retrieval in a microwave oven (300 W) in citrate buffer (pH 6.0) for 10 min at 100°C, the liver section then restored at room temperature and were sequentially preincubated with 1% BSA for 30 min at room temperature. They were then incubated with the primary antibody β-arrestin2 (dilution 1:100, Bioworld, USA), overnight at 4°C. After washing with phosphate-buffered saline (PBS), they were incubated with  a polymerized anti-rabbit immunoglobulin G (IgG) (dilution 1:200, Jingmei, Shanghai, China). Antibodies were visualized as brown granules in the cytoplasma using a DAB kit^39^ (Maixin Biological Technology, Fujian, China). Area density of β-arrestin2 positive tissues were analysed in 6 random high powered microscopic fields using Image-Pro-Plus® Software.

### Immunofluorescence analysis in cell culture

RAW264.7 cells were seeded in 24-well plate at 10^5^ cells/well, after 24 h incubated, cells were treated with 10 ng/ml remifentanil for 30 min.Then cells were washed twice with PBS and fixed with 4% paraformaldehyde, then blocked with 1% BSA. The fixed cells were then incubated with anti-β-arrestin2 antibody (dilution 1:100, Bioworld, USA) overnight at 4°C, washed in PBS for three times, and finally incubated with the second antibodies at room temperature for 2 h. DNA was stained with DAPI (diamidino-2-phenylindole) for 3 min and washed with PBS. The samples were then observed under an immunofluorescence microscope^40^.

### Western blot and immunoprecipitation

Cells were washed twice with ice-cold PBS and lysed in lysis buffer (20 mM Tris, pH 7.5, 150 mM NaCl, 1% Triton X-100, 1 mM phenyl methyl sulfonyl fluoride (PMSF), Beyotime) for 20–30 min on ice. If from frozen liver tissues, proteins were extracted by grinding with protease inhibitors.

Protein concentration was measured by the BCA assay (Beyotime, China). The proteins were resolved by sodium dodecyl-sulfate–polyacrylamide gel electrophoresis (SDS–PAGE) and then transferred to nitrocellulose filter (NC) membranes (Millipore, Bedford, MA). The membranes were blocked with 5% non-fat dry milk in 0.05% Tween-20–PBS for 2 h and incubated with the following primary antibodies: anti- β-arrestin2(Bioworld, USA), anti-TLR4(Abcam, USA), anti-pERK or anti-pJNK (Santa Cruz, USA)antibodies overnight at 4°C. The second antibody was combined with the appropriate horseradish peroxidase (HRP) and visualized by ECL detection kit (Millipore, USA). All the experiments reported in this study were repeated three times and the results were reproducible. For immunoprecipitation studies, cells were lysed at 4 °C for 1 h in cell lysis buffer for Western and IP containing 1 mM phenyl methyl sulfonyl fluoride (PMSF) from Beyotime^41,42^ (China). After centrifugation for 15 min at 12,000 g at 4 °C, soluble lysates were incubated overnight at 4 °C with 10 mg primary Abs prebound to protein A/G beads (Beyotime, China). Beads were pelleted and washed three times with lysiss buffer. Immunoprecipitated complexes were used for immunoblot as described above.

### Small interfering RNA and transfection

A day before siRNA treatment, RAW264.7 were seeded in 6-well plates at 5 × 10^5^ cells/well. After 24 h incubation, the cells were transfected with β-arrestin2 siRNA or scramble siRNA^43^ (Bioeasy, Shanghai, China). The interfering effect of the target by siRNA was confirmed by RT-PCR and Western blot.

### Quantitative Real-Time PCR (qRT-PCR)

Total RNA was isolated by using Trizol reagent (Invitrogen, Carlsbad, CA). Two micrograms of total RNA was used to synthesize first-strand cDNA using a RT-PCR kit (Invitrogen, Carlsbad, CA, USA). The primers of β-arrestin2 used in this study were as follows: forward 5-AGTCGAGCCCTAACTGCAAG-3, reverse 5-ACGAACACTTTCCGGTCCTTC-3. GAPDH was used as a control in a similar way using the following primers: forward 5-TGACCTCAACTACATGGTCTACA-3, reverse 5-CTTCCCATTCTCGGCCTTG-3.

### Measurement of cell viability assay and apoptotic cells

RAW264.7 cells were seeded in 96-well plates at 10^4^ cells/well. The plates were pretreated with 10 ng/ml remifentanil for 60 min and then incubated with 1 μg/ml LPS for another 24 h. Cell viability was detected with WST-8 dye^44^ (Beyotime Institute of Biotechnology, Jiangsu, China) according to the product reference. Cells were seeded in a 24-well plate at 10^5^ cells/well. Next, cells were pretreated with remifentanil for 60 min and then incubated with LPS for 6 h. After treatment, Cells were stained with Hoechst 33342 dye assay (Beyotime Institute of Biotechnology) for 15 min at room temperature after washed with saline for twice, and then examined with a fluorescent microscope.

Data analysis was performed with commercial software (Prism version 5.0; Graph-Pad software, San Diego, CA). All data were expressed as mean ± SD and were statistically analyzed by a one-way ANOVA. The Student-Newman-Keuls q test were used to compare values among all groups. Statistical differences significant level was performed at P < 0.05.

## Supplementary information

Supplementary Information
