# Supplementary material for: Indispensable role of β-arrestin2 in the protection of remifentanil preconditioning against hepatic ischemic reperfusion injury
Source: Sci Rep. 2019 Feb 14;9:2087. doi: 10.1038/s41598-018-38456-9 (PMC6376065; doi:10.1038/s41598-018-38456-9)

Supplementary material

Indispensable role of β-arrestin2 in the protection of remifentanil preconditioning against hepatic ischemic reperfusion injury

Yuting Yang[1]*, Caiyang Chen[1]*, Cui Cui[2]*, Yingfu Jiao[1], Peiying Li[1], Ling Zhu[1], Weifeng Yu[1], Qiang Xia[3], Daxiang Wen[1] & Liqun Yang[1]

1. Department of Anesthesiology, Renji Hospital, Shanghai Jiao Tong University School of Medicine
2. Department of Anesthesia and Intensive Care, Eatern Hepatobiliary Surgery Hospital, Second Military Medical University
3. Department of Hepatic Surgery, Renji Hospital, Shanghai Jiao Tong University School of Medicine

No.160 Pujiang Road, Shanghai, 200127, China

Tel. +86-21-86383456

* these authors contributed equally to this work. Correspondence and requests for materials should be addressed to Prof. Wen D (email:wdxrwj@126.com) or Prof. Yang L (email: lqyang72721@126.com)

Supplementary Figure Legend

Figure S1. Effects of remifentanil on the hepatocyte proliferation with hepatocyte: KC cocultures after hypoxia/reoxygenation injury (A-E). Remifentanil can also inhibit the expression of cytokines TNF-α and IL6 in cell culture supernatant(F-I). Cells were pretreated with remifentanil (10ng/ml) for 60 min, then then were incubated in an anaerobic chamber (Thermo Forma Inc., Marietta, OH) for 6 h and the fixed cells were reoxygenated for up to 12 h at 37°C (* P<0.05 compared to O2-/Ref- group). All the results were from at least three independent experiments.

Figure S1.

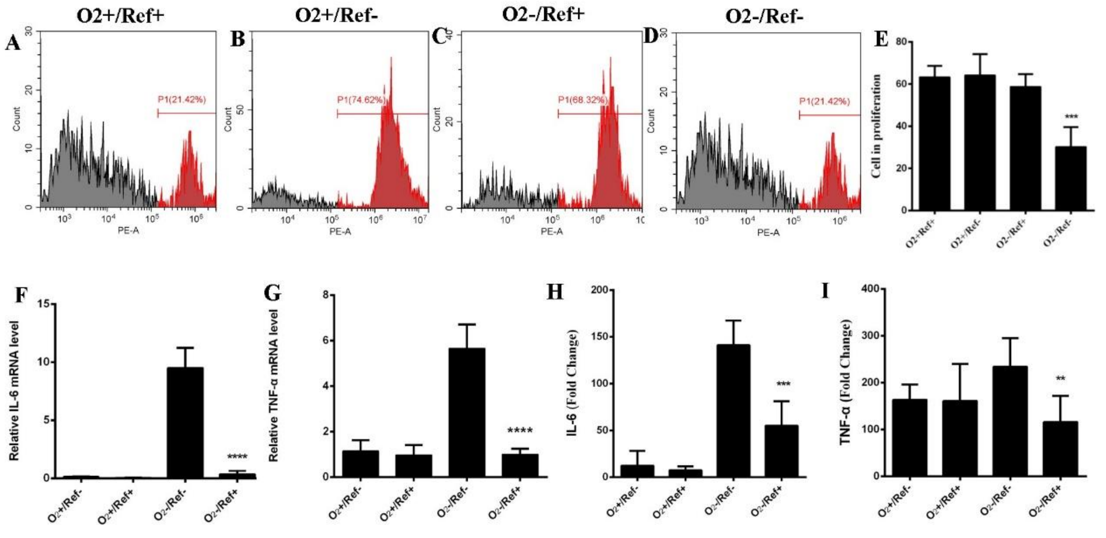

Supplement: Supplementary file 1 — Supplementary Information [file 41598_2018_38456_MOESM1_ESM.pdf]
